# Application of a Hybrid Method Combining Grey Model and Back Propagation Artificial Neural Networks to Forecast Hepatitis B in China

**DOI:** 10.1155/2015/328273

**Published:** 2015-02-26

**Authors:** Ruijing Gan, Xiaojun Chen, Yu Yan, Daizheng Huang

**Affiliations:** School of Preclinical Medicine, Guangxi Medical University, No. 22, Shuangyong Road, Nanning, Guangxi 530021, China

## Abstract

Accurate incidence forecasting of infectious disease provides potentially valuable insights in its own right. It is critical for early prevention and may contribute to health services management and syndrome surveillance. This study investigates the use of a hybrid algorithm combining grey model (GM) and back propagation artificial neural networks (BP-ANN) to forecast hepatitis B in China based on the yearly numbers of hepatitis B and to evaluate the method's feasibility. The results showed that the proposal method has advantages over GM (1, 1) and GM (2, 1) in all the evaluation indexes.

## 1. Introduction

Hepatitis B is a vaccine preventable disease caused by the hepatitis B virus (HBV) that can induce potentially fatal liver damage. It has infected approximately 2 billion people worldwide, which represents one-third of the world population. Each year around the world, HBV infection is responsible for about one million deaths due to liver failure and cirrhosis and more than 75% of the hepatocellular carcinomas world-wide develop from HBV infection [1–3]. HBV is most prevalent in China, South East Asia, sub-Saharan Africa, and the Amazon basin of South America where health care resources are most limited [4]. In the Chinese population of 1.3 billion individuals, there are estimated to be 93 million HBV carriers. Each year, 300,000 deaths are attributed to chronic hepatitis B, including deaths associated with liver cirrhosis and hepatocellular carcinoma (HCC) [5]. Therefore, it is critical for early prevention of hepatitis B and an accurate forecasting which would enable public health officials to evaluate intervention strategies and make educated decisions.

Mathematical and computational models have gained in importance in the public-health domain, especially in infectious disease epidemiology, by providing rationales and quantitative analysis to support decision-making and policy-making processes in recent years. And many researchers advocate the use of these models as predictive tools [6–12].

The accurate forecasting of hepatitis B can be obtained by analyzing the sufficient historical data. However, in China and perhaps some other developing countries, the current public health surveillance system does not collect detailed essential epidemiological information as they are often difficult to obtain. The forecasted of hepatitis B will be inaccurate only by the limited data. Therefore, it is significant to make the limited data-processing.

The grey systems theory chiefly including the theory of grey system analysis, modeling, prediction, decision-making, and control is established by Deng, which focuses on uncertainty problems with small samples, discrete data and incomplete information that are difficult for probability, and fuzzy mathematics to handle. Grey prediction is an important embranchment of grey systems theory, which makes scientific, quantitative forecasts about the future states of grey systems. The precise prediction of system can be performed by generating and extracting the useful information from the small samples and the partially known information [13–15].

Artificial neural networks (ANN) are complex and flexible nonlinear systems with properties not found in other modeling systems. It allows a method of forecasting with understanding of the relationship among variables and in particular nonlinear relationships. ANN function by initially learning a known set of data from a given problem with a known solution (training) and then the networks, inspired by the analytical processes of the human brain, are able to reconstruct the imprecise rules. Once a model is trained, the forecasted outputs can be generated from novel records [16–19].

The aim of this study is to investigate the use of a hybrid method combining grey model (GM) and back propagation artificial neural networks (BP-ANN) to forecast hepatitis B in China based on the yearly numbers of hepatitis B from the years 2002 to 2012 and to evaluate the method's performances of prediction.

## 2. Materials and Methods

### 2.1. Data Sources

The incidence data of hepatitis B are collected from the Ministry of Health of the People's Republic of China from the years 2002 to 2012, which are opening government statistics data [20].

### 2.2. Methods

The proposed method is established based on the grey systems theory and BP-ANN theory. MATLAB software version 2011b is used for the statistical analysis.

The incidence data are considered as the original time series *X* = (*x*_0_, *x*_1_, *x*_2_,…, *x*_*n*_), where *n* is the length of the time series.

Through grey generations or the effect of sequence operators to weaken the randomness, grey prediction models are designed to excavate the hidden laws; through the interchange between difference equations and differential equations, a practical jump of using discrete data sequences to establish continuous dynamic differential equations is materialized. Here, GM (1,1) is the main and basic model of grey predictions, that is, a single variable first order grey model, which is able to acquire high prediction accuracy despite requiring small sample size (but the sample size must be at least 4). The GM (1,1) model is suitable for sequences that show an obvious exponential pattern and can be used to describe monotonic changes. As for nonmonotonic wavelike development sequences, or saturated sigmoid sequences, one can consider establishing GM (2,1) model.

The establishment for a GM (1,1) model is derived as follows.

(1) Let nonnegative time sequence expressing *X* ^0^ = (*x* ^0^(1), *x* ^0^(2),…, *x* ^0^(*n*)) be an original time sequence. Where *n* is the sample size of the data.

(2) First-order accumulative generation operation (1-AGO) is used to convert *X* ^0^ into *X* ^1^ = (*x* ^1^(1), *x* ^1^(2),…, *x* ^1^(*n*)) = (∑_*i*=1_^1^ *x* ^0^(*i*), ∑_*i*=1_^2^ *x* ^0^(*i*),…, ∑_*i*=1_^*n*^ *x* ^0^(*i*))

(3) Let *Z* ^1^ = (*z* ^1^(2), *z* ^1^(3),…, *z* ^1^(*n*), ) be the sequence generated from *X* ^1^ by adjacent neighbor means. That is, *z* ^1^(*t*) = 0.5(*x* ^1^(*t*) + *x* ^1^(*t* − 1)), *t* = 2,3,…, *n*. The least square estimate sequence of the grey difference equation of GM (1,1) is defined as *x* ^0^(*t*) + *az* ^1^(*t*) = *b*, where −*a* and *b* are referred to as the development coefficient and grey action quantity, respectively.

Then

(1) $$\begin{array}{c} {\left[ab\right]}^{T}={\left(B^{T}B\right)}^{-1}B^{T}Y, \end{array}$$

where

(2) Yx02x03⋮x0n,B=−z121−z131⋮⋮−z1n1.

(4) The whitenization equation is given by *dx* ^1^/*dt* + *ax* ^1^ = *b*

(5) The forecasting model can be obtained by solving the above equation, which is shown as follows:

(3) $$\begin{array}{c} {\hat{x}}^{1}\left(t+1\right)=\left(x^{0}\left(1\right)-\frac{b}{a}\right)e^{-at}+\frac{b}{a}. \end{array}$$

(6) The predicted value of the primitive data at time point (*t* + 1) is extracted:

(4) x^0t+1x^1t+1−x^1t=1−eax01−bae−at.

The procedure for a GM (2,1) model is derived as follows.

(1) For a given sequence of original data *X* ^0^ = (*x* ^0^(1), *x* ^0^(2),…, *x* ^0^(*n*)), let its sequences of accumulation generation and inverse accumulation generation be *X* ^1^ = (*x* ^1^(1), *x* ^1^(2),…, *x* ^1^(*n*)) and *α* ^1^ *X* ^0^ = (*α* ^1^ *x* ^0^(1),…, *α* ^1^ *x* ^0^(*n*)), respectively, where *α* ^1^ *x* ^0^ = *x* ^0^(*t*) − *x* ^0^(*t* − 1)), *t* = 2,3,…, *n* and the sequence of adjacent neighbor mean generation of *X* ^1^ is *Z* ^1^ = (*z* ^1^(2), *z* ^1^(3),…, *z* ^1^(*n*), ).

(2) The GM (2,1) model is *α* ^1^ *x* ^0^(*t*) + *a*_1_ *x* ^0^(*t*) + *a*_2_ *z* ^1^(*t*) = *u* and the whitenization equation is given by *d* ^2^ *x* ^1^/*dt* ^2^ + *α*_1_(*dx* ^1^/*dt*) + *α*_2_ *x* ^1^ = *u*. The least squares estimate of the parametric sequence is

(5) $$\begin{array}{c} \hat{a}={\left[a_{1},a_{2},u\right]}^{T}={\left(B^{T}B\right)}^{-1}B^{T}Y, \end{array}$$

where

(6) Yα1x02α1x03⋯α1x0n=x02−x01x03−x02⋯x0n−x0n−1,B−x02−z121−x03−z131⋯⋯⋯−x0n−z1n1.

(3) Solve the whitenization equation. If *X* ^1∗^ is a special solution of the whitenization equation and ${\bar{X}}^{1}$ the general solution of the corresponding homogeneous equation *d* ^2^ *x* ^1^/*dt* ^2^ + *α*_1_(*dx* ^1^/*dt*) + *α*_2_ *x* ^1^ = 0, then $X^{1*}+{\bar{X}}^{1}$ is the general solution of the GM (2,1) whitenization equation. There are three cases for the general solution of the homogeneous equation: (i) when the characteristic equation *r* ^2^ + *α*_1_ *r* + *α*_2_ = 0 has two distinct real roots ${\bar{X}}^{1}=c_{1}e^{r_{1}t}+c_{2}e^{r_{2}t}$; (ii) when the characteristic equation has a repeated root ${\bar{X}}^{1}=e^{rt}(c_{1}+c_{2}t)$; (iii) when the characteristic equation has two complex conjugate roots *r*_1_ = *α* + *iβ*  and  *r*_2_ = *α* − *iβ*, ${\bar{X}}^{1}=e^{\alpha t}(c_{1}\cos\beta t+c_{2}\sin\beta t)$. A special solution of the whitenization equation may take of the three possibilities: (i) when 0 is not a root of the characteristic equation, *X* ^1∗^ = *C*; (ii) when 0 is one of the two distinct roots of the characteristic equation, *X* ^1∗^ = *Cx*; and (iii) when 0 is the only root of the characteristic equation, *X* ^1∗^ = *Cx* ^2^.

The steps of the forecasting method can be described as follows.

**Step 1 (train the BP-ANN).**

In order to obtain the input of the BP-ANN, the GM (1,1) and GM (2,1) model are used to predict for the original time series of hepatitis B, respectively. The two groups of predicted are taken as the input of the BP-ANN. At the same time, the original time series are taken as the output. Thus the structure of a three-layer BP-ANN is constructed and the trained BP-ANN model will be obtained by training.

**Step 2 (forecast by the trained BP-ANN).**

The GM (1,1) and GM (2,1) model are used to forecast for the original time series of hepatitis B, respectively, at first. Then the two groups of forecasted data are taken as the input of the trained BP-ANN. Finally, the forecasted of hepatitis B will be obtained by running the trained BP-ANN.

**Figure 1:**
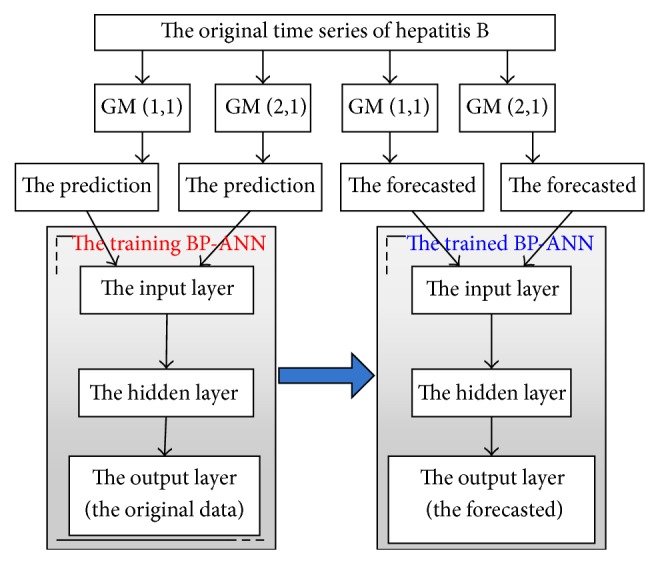
Flow chart of the hybrid method.

The method flow chart is shown in Figure 1.

## 3. Evaluation Criteria

The metrics used are relative error (RE), $\text{R}{\text{E}}_{i}=\left|{\hat{y}}_{i}-y_{i}\right|/y_{i}$, *i* = 1,2,…, *n*, where ${\hat{y}}_{i}$ is the forecasted data and *y*_*i*_ is the actual data; correlation coefficient (*R*), $R=R_{\hat{y}y}/\text{std}\left(y\right)\text{std}\left(\hat{y}\right)$, where $R_{\hat{y}y}$ is the covariance between *y*  and  $\hat{y}$; mean square error (MSE), $\text{MSE}=(1/n){\sum_{i=1}^{n}\left({\hat{y}}_{i}-y_{i}\right)}^{2}$; root mean square error (RMSE); $\text{RMSE}=\sqrt{(1/n){\sum_{i=1}^{n}\left({\hat{y}}_{i}-y_{i}\right)}^{2}}$; mean average error (MAE), $\text{MAE}=(1/n)\sum_{i=1}^{n}\left|{\hat{y}}_{i}-y_{i}\right|$; mean average percentage error (MAPE), $\text{MAPE}=(1/n)\sum_{i=1}^{n}\left|{\hat{y}}_{i}-y_{i}\right|*100$, and sum of squared error (SSE), $\text{SSE}={\sum_{i=1}^{n}\left({\hat{y}}_{i}-y_{i}\right)}^{2}$.

## 4. Result

**Figure 2:**
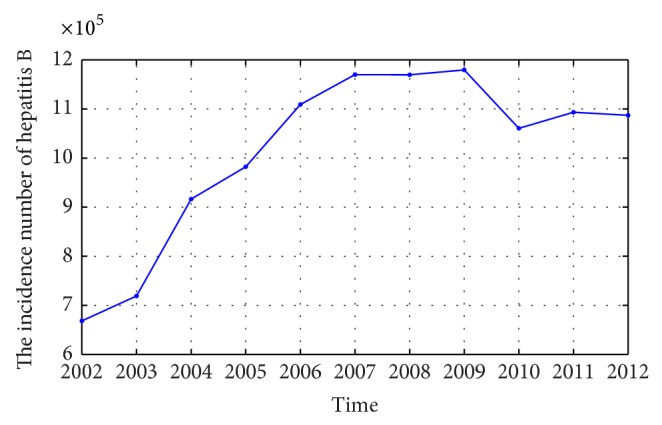
The incidence number of hepatitis B in China from 2002 to 2012.

The incidence data of hepatitis B are collected year by year from 2002 to 2012 in China and taken as the original time series, which is shown in Figure 2.

### 4.1. The GM (1, 1) and GM (2, 1) Models Calculation

The GM (1,1) and GM (2,1) models are calculated and shown as follows.

#### 4.1.1. The Parameters

*GM (1, 1) Model.* Consider the following: −*a* = 0.027523, *b* = 893075.402372. Therefore, the GM (1,1) model of this time series can be forecasted for long term forecasting for the reasons that GM (1,1) can be used for long term forecasting when −*a* ≤ 0.3 and for short term forecasting when 0.3 < −*a* ≤ −0.5. −*a* reflects the development states of the accumulation generated sequence ${\hat{x}}^{\left(1\right)}$ and the sequence of raw data ${\hat{x}}^{\left(0\right)}$.

*GM (2, 1) Model.* Consider the following: *a*_1_ = 0.4083, *a*_2_ = 0.0158, *u* = 583425.10336, *r*_1_ = −0.04329, *r*_2_ = −0.36502, *c*_1_ = −38764735.45099, and *c*_2_ = 2527585.93078.

#### 4.1.2. The Forecasting Models

*GM (1, 1) Model*. Consider the following:

(7) xt+1−33116202.802344e0.027523t+32447876.802344.

*GM (2, 1) Model*. Consider the following:

(8) x1t+1−38764735.45099e−0.04329t+2527585.93078e−0.36502t+36918146.77020.

### 4.2. The Forecasted

**Figure 3:**
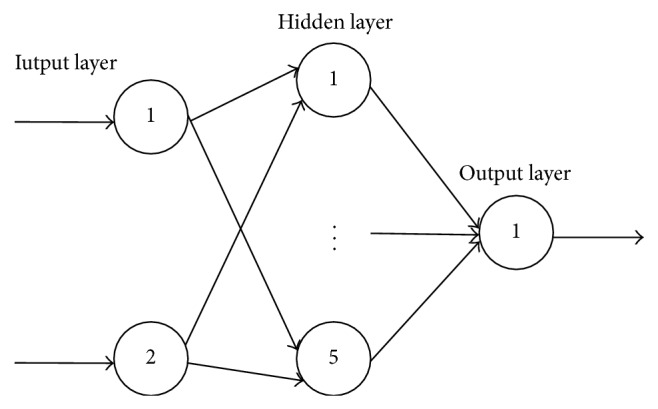
The topology structure of the proposal method.

In the three-layer BP-ANN, the hidden node *n*_2_ and the input node *n*_1_ are related by *n*_2_ = 2*n*_1_ + 1. The two groups of prediction created by the two GM models are taken as the input of the BP-ANN and the observed data is taken as the output. Therefore, a three-layer proposed model with 2 input nodes, 5 hidden nodes, and 1 output node is obtained. The topology structure is shown in Figure 3.

The weights and thresholds of the proposal model will be obtained by training. Let the training time be 1000, the learning rate be 0.9, the momentum factor be 0.95, and the error be 0.001; Levenberg Marquardt is used as training algorithm.

**Figure 4:**
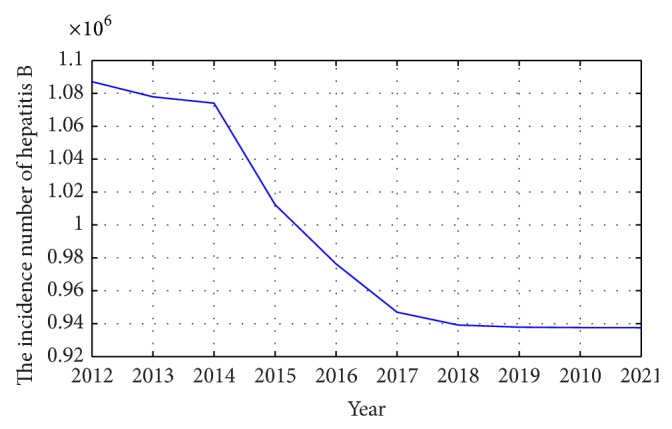
The forecasted incidence of hepatitis B in China from 2013 to 2021 by the proposal method.

The prediction of the original time series by the GM (1,1) and GM (2,1) model, respectively, are taken as the input of the trained BP-ANN. Then the forecasted of hepatitis B will be obtained by running the trained BP-ANN. The forecasted is shown in Figure 4.

## 5. Discussion

**Figure 5:**
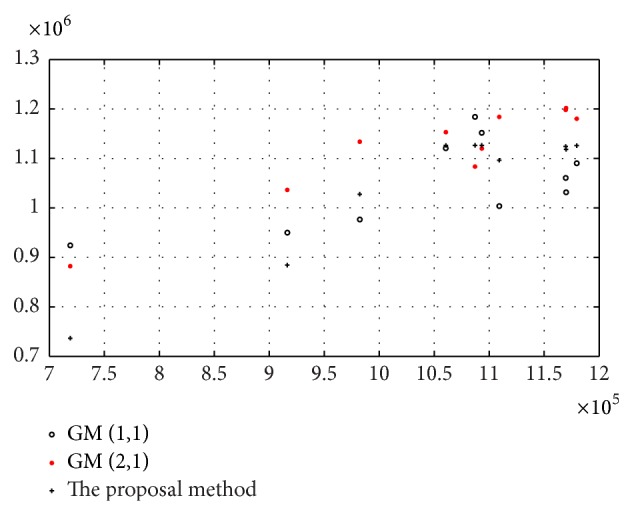
The scatter diagram of the relationship between the observed data and the prediction.

In order to compare the prediction created by the two GM models and the proposed method, a prediction is performed under the same conditions. The results are listed in Table 1 and the scatter diagram is shown in Figure 5. It can be seen from Figure 5 that the prediction generated by the two GM models has greater dispersion than that by the proposed method.

**Figure 6:**
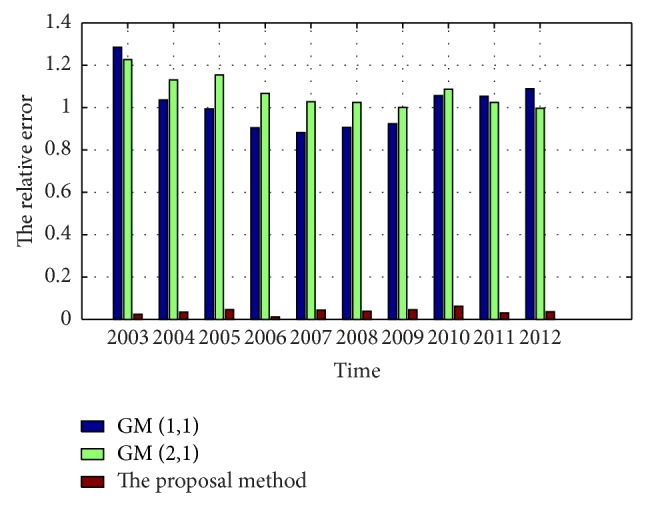
Comparison of the RE of the prediction by the proposal method and the GMs.

The RE of prediction is shown in Figure 6. From the figure, we know that the prediction obtained by the proposed method has higher accuracy and smaller RE than that by the GM approaches. Figure 6 indicates that the smaller the relative error is, the closer prediction is to the observed data.

The comparison of *R*, MSE, MAE, RMSE, MAPE, and SSE are listed in Table 2. It can be seen that the proposed method has advantages over GMs in all the evaluation indexes.

**Figure 7:**
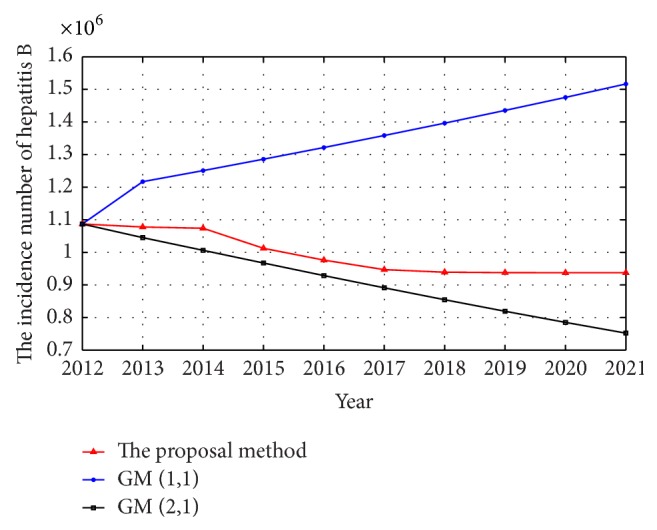
The forecasted incidence of hepatitis B in China from 2013 to 2021 by the three methods.

The forecasted generated by the GM (1,1), GM (2,1), and the proposal model are listed in Table 3 and shown in Figure 7.

**Figure 8:**
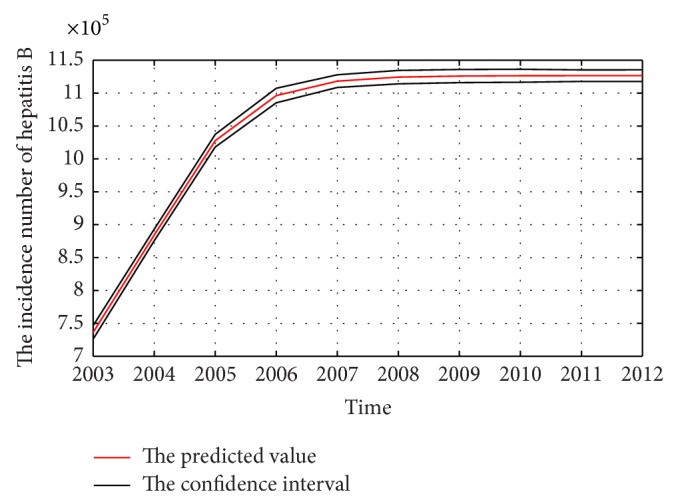
The predicted incidence of hepatitis B in China from 2003 to 2012 by the proposal methods.

**Figure 9:**
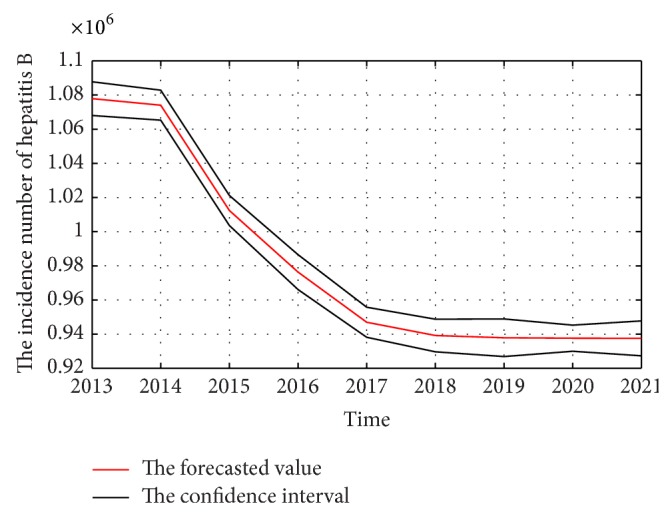
The forecasted incidence of hepatitis B in China from 2013 to 2021 by the proposal methods.

The weights and thresholds of BP-ANN will generate randomly at first when the model is training. This will make the predicted and forecasted uncertainty. To describe this clearer, the proposal model is ran 100 times and the mean value will be taken as predicted or forecasted value. The 95% confidence interval and predicted or forecasted value are shown in Figures 8 and 9, respectively.

Although the prediction result created by the proposal method in the paper has more accurate than that by the two gray models, the proposal model has its limitations. Firstly, since the proposal model is built on the basis of gray model, the sample size, namely, the number of historical data must be not less than 4. Secondly, the prediction result will be inaccuracy if the weights and thresholds in BP-ANN ran into local optimum in the process of training. Intelligent algorithms can be used to optimize the weights and thresholds of BP-ANN [21].

## 6. Conclusion

The hepatitis B epidemiological information is often difficult to obtain. Forecasting of hepatitis B will be inaccurate by the limited data. The grey systems theory focuses on uncertainty problems with small samples and incomplete information. At the same time, the BP-ANN is a method of forecasting with understanding of the relationship among variables and nonlinear relationships. The research proposes a new forecasting method, which combines the GM and BP-ANN, to forecast hepatitis B in China. The useful information can generate and extract from the small samples and the BP neural networks can train data more sufficiently. The prediction results show that this method can obtain better forecasting.

## Figures and Tables

**Table 1:** The prediction created by the GM (1, 1), GM (2, 1), and the proposal model.

Year	The observed data	GM (1, 1)	GM (2, 1)	The proposal method
2003	719011	924130	882183	736600
2004	916396	949918	1036306	884300
2005	982297	976426	1133758	1027700
2006	1109130	1003674	1183850	1096300
2007	1169946	1031682	1201811	1118200
2008	1169569	1060471	1198178	1124300
2009	1179607	1090065	1180237	1125900
2010	1060582	1120484	1153018	1126400
2011	1093335	1151751	1119982	1126500
2012	1087086	1183892	1083509	1126500

**Table 2:** The evaluation indexes comparison.

Index	*R*	MSE	MAE	RMSE	MAPE	SSE
The proposal method	0.9495	2.3649 × 10^7^	3.9704 × 10^4^	4.863 × 10^3^	3.9704 × 10^6^	1.8162 × 10^10^
GM (1, 1) model	0.6365	2.2867 × 10^8^	1.0492 × 10^6^	1.5122 × 10^4^	1.0492 × 10^8^	1.1078 × 10^13^
GM (2, 1) model	0.9392	1.6798 × 10^8^	1.1173 × 10^6^	1.5122 × 10^4^	1.1173 × 10^8^	1.2570 × 10^13^

**Table 3:** The forecasted generated by the three methods.

Year	GM (1, 1)	GM (2, 1)	The proposal method
2013	1216929.0	1045223.6	1077864.1
2014	1250888.2	1006228.6	1074038.2
2015	1285795.1	967267.6	1012371.7
2016	1321676.0	928834.0	976301.8
2017	1358558.3	891248.9	946959.7
2018	1396469.7	854715.0	939194.1
2019	1435439.1	819353.3	937881.5
2020	1475496.0	785229.0	937607.7
2021	1516670.7	752369.4	937531.5

## References

[1] Gourley S. A., Kuang Y., Nagy J. D. (2008). Dynamics of a delay differential equation model of hepatitis B virus infection. *Journal of Biological Dynamics*.

[2] Liu F., Chen L., Yu D.-M. (2011). Evolutionary patterns of hepatitis B virus quasispecies under different selective pressures: correlation with antiviral efficacy. *Gut*.

[3] Torpy J. M., Burke A. E., Golub R. M. (2011). JAMA patient page. Hepatitis B. *Journal of the American Medical Association*.

[4] Nwokediuko S. C. (2011). Chronic hepatitis B: management challenges in resource-poor countries. *Hepatitis Monthly*.

[5] (2011). The guideline of prevention and treatment for chronic hepatitis B (2010 version). *Chinese Journal of Hepatology*.

[6] Zhang T., Teng Z. (2007). On a nonautonomous SEIRS model in epidemiology. *Bulletin of Mathematical Biology*.

[7] Tizzoni M., Bajardi P., Poletto C. (2012). Real-time numerical forecast of global epidemic spreading: case study of 2009 A/H1N1pdm. *BMC Medicine*.

[8] Eubank S., Guclu H., Kumar V. S. A. (2004). Modelling disease outbreaks in realistic urban social networks. *Nature*.

[9] Ren H., Li J., Yuan Z.-A., Hu J.-Y., Yu Y., Lu Y.-H. (2013). The development of a combined mathematical model to forecast the incidence of hepatitis E in Shanghai, China. *BMC Infectious Diseases*.

[10] Bai Y., Jin Z. (2005). Prediction of SARS epidemic by BP neural networks with online prediction strategy. *Chaos, Solitons and Fractals*.

[11] Ture M., Kurt I. (2006). Comparison of four different time series methods to forecast hepatitis A virus infection. *Expert Systems with Applications*.

[12] Thornley S., Bullen C., Roberts M. (2008). Hepatitis B in a high prevalence New Zealand population: a mathematical model applied to infection control policy. *Journal of Theoretical Biology*.

[13] Shen X., Ou L., Chen X., Zhang X., Tan X. (2013). The application of the grey disaster model to forecast epidemic peaks of typhoid and paratyphoid fever in China. *PLoS ONE*.

[14] Deng J. L. (1989). Introduction to grey system theory. *The Journal of Grey System*.

[15] Liu S. F., Lin Y. (2010). *Grey Systems: Theory and Applications*.

[16] Amaritsakul Y., Chao C.-K., Lin J. (2013). Multiobjective optimization design of spinal pedicle screws using neural networks and genetic algorithm: mathematical models and mechanical validation. *Computational and Mathematical Methods in Medicine*.

[17] Zou J., Han Y., So S. S. (2008). Overview of artificial neural networks. *Methods in Molecular Biology*.

[18] Terrin N., Schmid C. H., Griffith J. L., D'Agostino R. B., Selker H. P. (2003). External validity of predictive models: a comparison of logistic regression, classification trees, and neural networks. *Journal of Clinical Epidemiology*.

[19] Grossi E., Buscema M. (2007). Introduction to artificial neural networks. *European Journal of Gastroenterology and Hepatology*.

[20] The Science Data Center of Public Health of China http://www.phsciencedata.cn/Share/ky_sjml.jsp?id=8defcfc2-b9a4-4225-b92c-ebb002321cea&show=0.

[21] Huang D., Gong R., Gong S. (2015). Prediction of wind power by chaos and BP artificial neural networks approach based on genetic algorithm. *Journal of Electrical Engineering & Technology*.

